# AI Applications in Electrocardiography for Ischemic and Structural Heart Disease: A Review of the Current State

**DOI:** 10.3390/jcm15010316

**Published:** 2026-01-01

**Authors:** Eugene J. Kim, Dhir Gala, Mohammed Ayyad, Manaal Pramanik, Amgad N. Makaryus

**Affiliations:** 1 Department of Medicine, Rutgers New Jersey Medical School, Newark, NJ 07003, USA; eugene.jay.kim@rutgers.edu (E.J.K.); dhirgala@gmail.com (D.G.); mohammed.ayyad@rutgers.edu (M.A.); 2 W. Tresper Clarke School, Salisbury, NY 11590, USA; manaalpramanik@gmail.com; 3 Department of Cardiology, Cardiovascular Institute, Northwell Health, New Hyde Park, NY 11040, USA; 4 Donald and Barbara Zucker School of Medicine at Hofstra/Northwell, Hempstead, NY 11549, USA; 5 Plainview Hospital, Northwell Health, Plainview, NY 11803, USA

**Keywords:** artificial intelligence, AI, deep learning, electrocardiography, STEMI, NSTEMI, OMI, cardiology, hypertrophic cardiomyopathy, aortic stenosis

## Abstract

Cardiovascular disease is the leading cause of morbidity and mortality worldwide, with ischemic and structural heart diseases being key contributors. While the 12-lead electrocardiogram (ECG) is a common low-cost diagnostic test, its interpretation is limited by human variability. Through machine learning with large diverse ECG data sets and artificial intelligence (AI) algorithms, ECG analysis can be automated for pattern recognition with higher accuracy. AI-augmented ECG algorithms have been demonstrated to be able to detect myocardial infarction with high accuracy and reduce door-to-balloon coronary intervention times. Similar models can be utilized to detect subtle ECG waveforms suggestive of current or future asymptomatic left ventricular dysfunction, aortic stenosis, and hypertrophic cardiomyopathy. Despite these promising results, there is concern for generalizability and bias or errors in training data. As AI systems evolve to multimodal integration, AI-augmented ECG has the potential to redefine cardiovascular diagnostics and enable earlier detection, risk stratification, and precision-guided interventions.

## 1. Introduction

Cardiovascular disease (CVD) remains the leading cause of death worldwide, accounting for an estimated 17.9 million deaths in 2019 [1,2]. Amongst CVDs, ischemic and structural heart diseases remain among the world’s leading causes of death and disability. Approximately 8.9 million deaths worldwide are attributed to ischemic heart disease (IHD). IHD and screening for ischemia or subtle structural abnormalities could allow for earlier intervention, risk reduction, and optimized target monitoring, all of which significantly lower mortality and morbidity [3]. For example, early valve intervention in asymptomatic aortic stenosis (AS) reduced the rate of New York Heart Association (NYHA) class II-IV symptoms at 12 months (19.7% vs. 37.9%; odds ratio 0.37; 95% CI 0.20–0.70) [4].

The 12-lead electrocardiogram (ECG) is the cornerstone of cardiovascular diagnostics. It is a low-cost, ubiquitous, and rapidly performed test that captures the heart’s electrical activity and myocardial perfusion, making it central to diagnosing a range of CVDs, especially acute coronary syndromes (ACS) [5]. Classic ischemic signs such as ST-segment changes, T-wave inversions, and pathologic Q waves enable early recognition of myocardial ischemia (MI) or infarction, facilitating rapid triage and expedited intervention. As early analog ECG systems transition to modern digital multichannel ECG platforms, enabling rapid transmission and standardized signal acquisition, the sheer volume of data presents new challenges [6,7]. Healthcare, cardiology in particular, is experiencing a data overload; imaging, electrophysiology tracings, continuous monitoring signals, and electronic health record (EHR) data are accumulating at rates beyond human-scalable review [8]. Each hospitalized cardiac patient can generate up to 1000 data points per second from continuous ECG monitoring, which can amount to terabytes of raw data daily in a single intensive care unit [9]. While experts can interpret individual ECGs or echocardiography (ECHO) studies, the integration of different data (e.g., combining ECG signals, metrics, clinical labs, imaging, and other trends) presents enormous logistical burdens [10].

Computational aids (such as computerized ECG interpretation algorithms) have existed since the 1960s; by 2006, an estimated 100 million ECGs in the United States (US) were being interpreted by computerized rule-based algorithms annually [11]. Medicare briefly ceased reimbursement for physician ECG over-reads due to confidence in automated interpretation, but quickly reversed this decision. While these systems did provide some benefit by decreasing analysis time by up to 24% to 28% for experienced readers, they were disturbingly inaccurate with abnormal rhythms, conduction, and abnormal wave form [12,13]. Despite limitations in early systems, especially in adaptability, complex patterns, and manufacturer-dependent performance, they became ubiquitous in healthcare and have augmented human ECG reading since [14,15]. Artificial intelligence (AI)—broadly encompassing machine learning (ML) and deep learning (DL) methods—has progressively entered healthcare domains over the past two decades [16,17,18,19]. Early uses included diagnostic image classification, predictive modeling for outcomes, and decision support. In cardiology, the nature of patterns in ECG and cardiac imaging makes them especially accommodating to ML-based pattern recognition [20].

Despite advances in diagnostics and treatment, early detection and risk stratification remain major challenges due to the overwhelming amount of data that physicians need to integrate. As AI systems are now on the precipice of broader adoption in clinical care, they promise to revolutionize early detection and risk stratification. This review examines the current applications and rapidly expanding evidence on AI implementation in ECG for ischemic and structural heart disease. This review summarizes the historical and current roles of ECG and ECHO in ischemic and structural cardiology, surveys AI/ML and deep learning approaches that have been applied to IHD (detecting occlusion, infarction patterns, risk prediction from ECG), and discusses practical limitations and implementation challenges that must be addressed before broad deployment.

## 2. AI in ECG Analysis

Artificial intelligence (AI) refers broadly to computer systems capable of performing tasks that typically require human intelligence [21]. Within AI, machine learning (ML) describes algorithms that can learn from data presented to it to identify patterns and make predictions without explicit programming [22]. A subset of ML, DL employs multilayered neural networks that autonomously learn hierarchical representations of complex data, providing an increasing complexity of data processing [23].

The history of AI and deep learning in cardiology dates back to the late 1980s, when foundational neural network algorithms such as backpropagation and early convolutional architectures laid the groundwork for modern pattern recognition [24,25]. In cardiology, two principal deep learning architectures dominate. Recurrent neural networks (RNNs) and their gated variants, such as long short-term memory (LSTM) networks, specialize in sequential, time-dependent data, allowing dynamic modeling of the cardiac cycle, continuous ECG monitoring, and physiologic time-series prediction [26]. On the other hand, convolutional neural networks (CNNs) are excellent for cardiology as they excel at analyzing spatially structured inputs, such as 2D ECHO frames, CT and MRI slices, or 1D temporal ECG signals, by intertwining local filters to capture morphological features [27]. CNNs, initially designed for image recognition, have significantly improved detecting arrhythmias, myocardial infarction, and structural heart abnormalities from raw ECG data [28,29,30]. Compared to traditional computerized ECG STEMI criteria, an occlusive myocardial infarction (OMI) AI-ECG model improved performance with most parameters comparable to and some even better than ECG experts [31]. 

## 3. Clinical Application and Studies

### 3.1. STEMI

Current computerized algorithms to detect STEMI vary greatly in sensitivity and specificity (0.62–0.93 and 0.89–0.99, respectively). AI-enhanced ECG analysis can aid in significantly improving their accuracy and precision [32,33]. Chang et al. developed a bidirectional, four-layer LSTM AI model to detect acute ST-elevation MI (STEMI) and achieved an AUROC of 0.98 in an external test set, outperforming cardiologists (0.898), emergency physicians (0.820), internists (0.765), and commercial algorithm (0.845). However, limiting the study’s utility was the lack of diversity of STEMI to train the model on and a lack of verification of STEMI with coronary angiography [34]. Regardless, they demonstrated a likely benefit in not just improving the current computerized algorithms but also improving the accuracy of the triage process.

Prompt diagnosis of MI is critical as every 30 min delay in treatment raises 1-year mortality by 7.5%. Using AI-enhanced ECG analysis to shorten symptom-to-needle time is therefore essential [35,36]. Reducing door-to-balloon time has been a priority and AI may play a role. Utilizing Chang et al.’s model, Wang and colleagues deployed a modified model—incorporating CNN into Chang’s LSTM AI model—to assess whether AI-ECG can be utilized to reduce door-to-balloon time by facilitating the triage of chest pain in the Emergency Department (ED). ECGs interpreted in real time by the AI-STEMI model, with automatic alerts for high-risk tracings sent to ED physicians and on-call cardiologists who determined cardiac catheterization laboratory (CCL) activation, were compared with a conventional group in which ED physicians interpreted ECGs without AI support and contacted cardiologists. The model performed well with door-to-balloon time reduced from 64.5 ± 35.3 min to 53.2 ± 12.7 min (*p* = 0.007), with 98.5% vs. 87.2% (*p* = 0.009) of door-to-balloon times being less than 90 min in the AI group vs. the conventional group [37]. Thus, AI-ECG decreased the front-end delay in recognizing STEMI by providing instant ECG interpretation, flagging high-risk atypical presentations, and paging the CCL team via automated alerts.

While much effort has been made to reduce door-to-balloon time, the prehospital delay can be one of the lengthiest time delays in this process. Building on Chang et al.’s work, Chen et al. incorporated CNN into Chang’s LSTM model and implemented an all-day AI-based triage system and a prehospital portable ECG device in an ambulance service to detect STEMI [38]. Chen et al.’s CNN-LSTM model interpreted 21,035 ECGs, identifying 213 cases of STEMI. Of these, 80.3% were confirmed to be STEMI and 19.7% were judged to be false positives by cardiologists evaluating the ECG, high sensitivity troponin-I levels, and coronary angiography. Four ECGs were false negatives (false-negative rate of 0.1%) with three due to similarity to early repolarization and one with hyperacute T waves in precordial leads. The model had an accuracy of 0.992, precision of 0.889, specificity of 0.994, and area under the receiver operating characteristic curve of 0.914. Furthermore, the AI’s response time of 37.2 ± 11.3 s was a magnitude shorter than the physician response time of 113.2 ± 369.4; physician response times were sometimes almost 10 min. Notably, Chen et al. used a disposable single-piece 12-lead ECG that likely decreased placement error and increased ease of use, leading to improved input data to the AI-ECG model [38]. Several limitations exist in this study; only 10 of the AI-STEMI model patients underwent coronary angiography, and there was limited diversity in data sets for training and validation, which likely contributed to the false-positive rate of 19.7%, and there is a need for a larger test population to further evaluate AI’s efficacy in this setting.

Herman et al. performed a registry retrospective analysis of 1032 patients with suspected STEMI that triggered cardiac catheterization laboratory (CCL) activation across three medical centers in the US (Sacramento, Houston, Boston). His group evaluated the performance and operational impact of AI-ECG for STEMI triage. The reference standard was angiographically confirmed lesion with positive cardiac enzymes. Compared to current standard triage, AI-ECG outperformed standard triage in detecting angiographically confirmed STEMI with an AUC of 0.937, sensitivity of 0.92 vs. 0.71, and specificity of 0.81 vs. 0.29. Interestingly, Herman et al.’s model also showed a reduced false-positive rate (7.9% vs. 41.8%), suggesting model and data set training can overcome the false-positive rates seen in Chen et al.’s model. Herman et al.’s model, however, had poor performance with subendocardial ischemia, post-cardiac arrest changes, and Takotsubo cardiomyopathy [39]. A primary limitation of this study is its retrospective registry design, and therefore, the analysis was unable to strictly control for baseline characteristics or unmeasured confounders that a randomized prospective trial could address. Furthermore, the model demonstrated specific diagnostic blind spots with poor performance in subendocardial ischemia, post-cardiac arrest patterns, and Takotsubo cardiomyopathy. Note that the study by Herman et al. is retrospective; this limits causal inference and prevents firm conclusions about the clinical utility or impact of the model in practice. In addition, training exclusively on OMI-confirmed cases introduces spectrum bias; when applied to a broader population that includes non-OMI presentations, performance (sensitivity/specificity) may vary but would be a more accurate reflection of real-world conditions.

The Artificial Intelligence-Powered Rapid Identification of ST-Elevation Myocardial Infarction via Electrocardiogram (ARISE) trial attempted to evaluate AI’s efficacy in diagnosing STEMI in the Emergency Department (ED). The primary endpoint was door-to-balloon time and secondary endpoints included incidence of new-onset low ejection fraction, cardiac death, and all-cause mortality. AI-ECG demonstrated a 17% reduction in median door-to-balloon time with the AI-ECG group having a median of 82 min (interquartile range, 62.5 to 89.5) vs. 96 min for the control group (interquartile range, 78 to 137). Impressively, the negative predictive value (NPV) was 99.9% and positive predictive value (PPV) was 89.5%. Of note, the AI-ECG group interquartile range was significantly narrower compared to the control group, likely contributing to reduced cardiac death in the intervention group vs. control (odds ratio, 0.73; *p* = 0.029) [40]. With only 77 in the intervention group and 68 in the control group, the study had few events analyzed despite the large sample randomized. Further limitations include that the analysis was not an intention to treat, potentially favoring the intervention group, and that it was a single-center randomized clinical trials (RCT). Further large multicenter RCT with angiographic confirmation will be needed in more generalized populations to verify the efficacy of implementing AI-ECG for STEMI triaging.

An example deployment of an AI-ECG model with low false-positive/high true-positive rates depends on the validation of the model in the local population of the healthcare system. If validated for the local population with coronary angiographic confirmation, deployment of a Two-Tier Activation could be used. Tier One would be used for high-probability STEMI cases. Prehospital ECG acquired by Emergency Medical Technicians or ED technicians could be automatically uploaded to the AI engine with some clinical context such as symptoms, paced rhythm, and post-cardiac arrest. If the AI engine returns a STEMI probability of greater than 95%, automatic CCL activation will alert the ED physician, cardiologist, interventionalist, and CCL staff with a cardiologist able to verify or rescind CCL activation.

Tier Two would be utilized if STEMI probability is 80–95%. In this scenario, the AI-ECG model could push an alert to the ED physician, on-call cardiologist, and interventionalist, showing the ECG, highlighting the concerning waveform and confidence level, and alerting the CCL charge nurse to place staff on standby. The on-call cardiologist or interventionalist could then confirm or deny CCL activation. This is just one example of how an AI-ECG model could be deployed to increase door-to-balloon time and minimize harm. Table 1 Summarizes the performance of multiple AI-ECG models for detecting ischemic heart disease.

### 3.2. NSTEMI

While STEMI is important, approximately 30% of patients with occlusion on angiography do not have ST-segment elevation, with NSTEMIs presenting a challenge in diagnosis for clinicians and deep learning models [35]. In a retrospective cohort study, Gustafsson et al. examined a deep learning model’s ability to discriminate STEMI and NSTEMI in 492,226 ECGs in 214,250 patients from Stockholm, Sweden. Their CNN-based AI-ECG model could discriminate STEMIs and NSTEMIs from controls with an AUROC of 0.991/0.832 and Brier score—a measure of how close predictions are to reality with 0 representing perfect accuracy—of 0.001/0.008. Furthermore, inspection of Grad-CAM plots showed that the model focused on nuances beyond the ST segment. For STEMI, the model focused on the down-sloping last part of the T wave; for NSTEMI, the model utilized the last part of the PQ segment and last part of the T wave. These results not only indicate AI-ECG’s ability to discriminate NSTEMI but also suggest its ability to detect more nuanced features [41].

The current ACS classification of STEMI vs. NSTEMI is being called into question and the landscape is trending towards OMI [42,43]. Herman et al. developed and evaluated an AI model, dubbed the Queen of Hearts AI model, to detect acute OMI on a single 2.5 s standard 12-lead ECG in international testing data of 2222 patients. Only using ECG waveforms as input, the AI model significantly outperformed the STEMI criteria with a sensitivity of 80.6% versus 32.5%, specificity of 93.7% vs. 97.7%, and AUC of 0.938 vs. 0.651. Furthermore, accuracy in detecting OMI was similar between the AI model and ECG experts (90.9% vs. 90.8%). Time to diagnosis of OMI was significantly shorter in the AI model when compared to STEMI criteria (2.3 h versus 5.3 h, *p* < 0.001), but similar to the ECG experts in the study (2.9 h, *p* = 0.08). These results likely stem from an optimized model being trained on a large number of angiographically confirmed ECGs [31]. While promising, the Queen of Hearts AI model study has several limitations that must be emphasized. The primary flaw was selection bias with all patients from ACS databases, with a high prevalence of OMI in these data sets. In addition, there is a lack of prospective validation, limited generalizability to asymptomatic patients, limited generalizability to women and the younger population, and potential conflicts of interest with the lead author being the co-founder and Chief Medical Officer of Powerful Medical.

### 3.3. Left Ventricular Dysfunction (LVD)

Estimated prevalence of asymptomatic left ventricular dysfunction (LVD) is between 1.4 and 4% of the population, with a higher prevalence in those with diabetes, hypertension, and the elderly [44,45,46]. Currently, there are no cost-effective noninvasive screening tools available for asymptomatic LVD. While studies on the use of N-terminal pro-B-type natriuretic peptide (NT-proBNP) have been disappointing, the use of AI-ECG as a screening tool is being evaluated given ECG’s low cost, ubiquity, and standardization [47,48,49].

Attia et al. trained a CNN AI model on ECGs from 35,970 patients that had a transthoracic ECHO performed within a 2-week interval of the ECG (89% performed within 24 h) to determine AI-ECG’s efficacy as an asymptomatic LVD screening tool. Choosing a cut-off of ejection fraction < 35%, area under the curve was 0.93 and sensitivity, specificity, and accuracy were 86.3%, 85.7%, and 85.7%, respectively. The positive predictive value (PPV) was disappointing at 35% but noted by the authors to be due to the EF cut-off of 35%; however, PPV was improved to 63.4% when an EF of <50% was chosen as the cut-off. Of the false positives from the EF < 35% cut-off, 59% had an EF between 35 and 49%, meaning that the ECG detected mild-to-moderate systolic LVD. While a cut-off of <35% reduces false-positive rates, AI-ECG’s utility as a screening tool is reduced since mild-to-moderate LVD will not be flagged despite the AI-ECG model able to detect LVD. Notably, the 5.8% false positives but with normal EF on TTE were found to have a four-fold increased risk of developing left ventricular dysfunction at 5 years (10% risk at 5 years). The high hazard ratio (HR) of false-positive patients and the 59% of false positives that had mild-to-moderate LVD suggests that the AI model is capable of subtle LVD including early subclinical dysfunction that exists before the EF is affected [50].

Attia et al. proceeded to externally validate the AI-ECG model in a prospective workflow for an EF < 35% in 16,056 adult patients (LVD prevalence of 7.8%) from Mayo Clinic ECG laboratory. Accuracy was 86.5% with a sensitivity and specificity of 82.5% and 86.8%, respectively. False positives (474) were evaluated and it was found that 39.8% had an EF between 36% and 49%. The positive likelihood ratio (LR) was 6.25, with a negative LR of 0.20; however, the positive LR is likely elevated due to the high prevalence. While the use of a NT-proBNP alone as a screening tool was disappointing in previous studies, Attia et al. found that the use of pro-BNP and AI-ECG with a cut-off of 125 pg/mL resulted in fewer than five false positives with no loss of true positives [51]. Attia et al. performed another external validation study utilizing the same AI-ECG model with an EF cut-off of 35%. Instead of Mayo Clinic patients, the study was performed on Know Your Heart study subjects which comprised adults living in two cities in Russia. While the AUC was 0.82, sensitivity and specificity were 26.9% and 97.4%, respectively. NPV was 99.5%—likely due to the prevalence of LVD being 0.6%—with an accuracy of 97% [52].

These initial validation tests suggest that further investigations into AI-ECG in conjunction with biomarker efficacy to rule out asymptomatic LVD should be evaluated. Notably, the high hazard ratio for future LVD in patients classified as “false positive” by AI-ECG despite normal TTE raises an important possibility: what we currently call *false positives* may in fact represent early electrical or structural dysfunction not yet detectable by echocardiography. A parallel can be drawn to heart failure staging, where Stage A denotes individuals *at risk* despite preserved imaging suggesting that AI-ECG abnormalities may serve as an early risk signal rather than a diagnostic error. If validated, such findings could justify classifying these patients into an at-risk subgroup, warranting stricter risk-factor modification and closer clinical surveillance.

In this sense, when asked what a “false positive” means clinically, the answer may be either no abnormality or an early dysfunction that our current tools simply cannot yet visualize; a distinction requiring dedicated longitudinal research. For patients with subtle AI-ECG findings suggestive of LVD, it may be reasonable to consider tightening risk-factor control and arranging periodic follow-up imaging, particularly when AI-ECG abnormalities persist or coexist with cardiovascular risk. Although we are not advocating for AI-ECG screening in the general population, its potential utility may be greatest among patients with known risk factors (e.g., CAD, HTN, diabetes, CKD, valvular disease, cardiomyopathy exposure), where prognostic enrichment is highest.

Still, given the high false-positive rates and the absence of prospective validation in diverse populations, changes in management should remain conservative, favoring observation and research rather than intervention. Nonetheless, the concept that AI-ECG may detect disease before imaging does is compelling and represents an avenue that warrants prospective study to determine whether early detection improves outcomes and resource allocation. The second external validation with a Russian population by Attia et al. brought into light a limitation of AI-ECG: spectrum bias. Similarly to other diagnostic tests, AI algorithms cannot be simply “dropped in” to new patient populations and will require recalibration prior to deployment into new settings.

Bias in AI algorithms has been a growing concern, given that the rise of AI and the training data collected could introduce inherent bias into the model [53,54,55,56]. Further investigation into AI-ECG detection of LV systolic dysfunction (LVSD) and bias was performed by Yagi et al. The goal of the study was to evaluate the performances of four de novo models, trained with different data sets from differing regional populations, in detecting LVSD, and to externally validate these AI-ECG models. Four de novo AI-ECG models were each trained with separate regional population ECG sets. While the Massachusetts General Hospital (MGH) model had good internal and external validity (AUROC 0.914 and 0.905, respectively) the Keio University Hospital model had good internal but poor external validity (AUROC 0.914 and 0.856, respectively). The results highlight the importance of external validation testing prior to deployment of AI-ECG models in a specific population as training on different data sets produces models with different performances. Highlighted by all models was the reduced detection in atrial fibrillation (AF), left-bundle branch block (LBBB), and paced rhythm, suggesting that stratification analysis is critical to determining AI-ECG model weaknesses prior to deployment and avenues for improvement [57]. Given the risk of spectrum bias and uncertainty around generalizability, institutions adopting AI-ECG systems should perform local validation and calibration testing before permitting management changes to ensure that performance is appropriate for the specific population they serve. Importantly, AI output should augment rather than replace physician interpretation; an expert over-read remains essential, particularly in early phases of implementation. The value of AI-ECG might most appropriately be to detect subtle patterns that may be missed, accelerating interpretation during high-acuity triage, and supporting fellows, residents, and community clinicians who may lack immediate access to expert input.

At the same time, reliance on AI confers new risks. A system trained on OMI-enriched data sets may underperform when faced with lower-prevalence real-world cases, and false reassurance from an apparently normal AI interpretation could be harmful, particularly if users are unaware of its blind spots. Over-reliance may also encourage cognitive offloading, reducing active ECG interpretation and clinical reasoning—potentially dulling the diagnostic skills AI is meant to enhance. Thus, education on limitations, failure modes, and human-in-the-loop oversight is critical to ensure implementation improves safety rather than inadvertently increasing diagnostic error.

König et al. performed an external validation of Yagi et al.’s AI-ECG model for detecting LVSD in a German population. Results were slightly worse than Yagi et al.’s results, with an AUROC for LVSD of 0.88 and sensitivity and specificity of 82% and 77%, but a negative predictive value of 96%. These results further highlight, similar to Yagi et al.’s study, the need for AI-ECG models to be trained on the population of interest and the need for external validation testing in the population of interest. Interestingly, the increased HR of 4 for some false positives remained consistent in the German population, further giving evidence of subtle subclinical ECG waveforms predictive of developing LVSD [58]. If AI-ECG is to be used to screen for asymptomatic LVD, these studies highlight the need for a larger and more diverse training data set that should include multiple ethnicities, regional populations, and inpatient and outpatient environments. Table 2 summarizes performance of AI-ECG and EHR-based models for detecting left ventricular dysfunction.

### 3.4. Structural Heart Disease

Life expectancy globally has been on the rise, with the population > 80 years of age projected to triple from 11.4 million in 2010 to 32.4 million in 2050 in the United States, with a larger percentage increase expected in developed countries [59]. The prevalence of valvular heart diseases (VHD), like life expectancy, will be expected to increase as its prevalence increases with age due to degenerative changes [60,61]. With mitral regurgitation (MR) and aortic stenosis (AS) the most prevalent valvular heart disorders and transcatheter or surgical therapeutics available, identification and screening have become more important. The rise of AI and its integration with ECG will play a role in the future for valvular heart disease screening.

AS begins with a long asymptomatic period; however, once symptomatic, mortality increases, with 40 to 50% of patients with classic symptoms dying in 1 year [62]. In patients with asymptomatic AS, good follow-up and close monitoring can yield good results with urgent aortic valve replacement when symptomatic [63,64,65]. Screening for asymptomatic AS would be important; however, no suitable screening tools currently exist. AI-ECG may one day fill that role as a low-cost ubiquitous screening tool for AS. Kwon et al. developed a multilayered CNN DL model to detect significant AS using AI-ECG. They tested both 12-lead AI-ECG and single-lead AI-ECG and found the internal and external validation AUROC for the 12-lead ECG to be 0.884 and 0.861. Impressively, the single-lead ECG internal and external validation AUROC was an impressive 0.845 and 0.821, respectively. Analysis found that the AI-ECG model mainly focused on the precordial (V1-4) T-wave axis, age, and QT interval with higher T-wave peaks and less frequent T-wave inversion in AS data groups [66].

Cohen-Shelly et al. developed a similar model to Attia et al. but to detect moderate-to-severe AS. Using 129,788 ECG–TTE-paired patients for training, they internally validated their model retrospectively within 25,893 patients and tested on 102,926 randomly selected subjects. Their model performed well solely on ECG with an AUC of 0.85 and sensitivity, specificity, and accuracy of 78%, 74%, and 74%, respectively. However, when other information such as age and sex data was added to the model, the AUC increased to 0.87. False-positive samples, like Attia et al.’s model, showed a HR increase of 2.18 in risk for developing moderate-to-severe AS in 15 years. The results suggest that AI-ECG models perform well for screening purposes for moderate-to-severe AS, can be improved with the integration of basic clinical data, and subtle ECG changes can provide future risk predictions [67]. AI-ECG performance was similar for mitral regurgitation, mitral stenosis, and pulmonary hypertension, including increased future risk in those who were false positive on AI-ECG but negative on TTE [68,69,70]. Table 3 summarizes performances of multiple AI-ECG models and AI-ECG with EHR-based models for detecting structural heart disease.

### 3.5. Hypertrophic Cardiomyopathy

Hypertrophic cardiomyopathy (HCM) is estimated to have a prevalence of 1 in 200 to 1 in 500, is the second most common type of cardiomyopathy, and is the most common cause of sudden cardiac death in young people, with the highest risk in younger patients [71,72]. Diagnosis traditionally relies on imaging with TTE or cardiac magnetic resonance imaging (cMRI) with family genetic screening of mutations of sarcomere protein genes indicated for first-degree relatives diagnosed with HCM. As new therapies for HCM, such as cardiac myosin inhibitors, enter the market, screening may become more important [73,74]. Current guidelines recommend only screening for first-degree relatives of affected individuals; however, with AI-ECG, that may change [75].

Ko et al., working with Attia, developed a CNN model for the detection of HCM which performed impressively well. AUC on an internal validation data set was 0.96 with a sensitivity and specificity of 87% and 90%, respectively, and performance improved in younger patients with a sensitivity of 95% and specificity of 92% [76]. This was followed up with a diverse international cohort external validation study and an AUC of 0.922, accuracy of 86.9%, sensitivity of 82.8%, and specificity of 87.7% were achieved [77]. While promising results, large-scale prospective evaluations of AI-ECG models are still needed. While much research has focused on AI-ECG, Sammani et al. developed a text-mining ML algorithm that evaluated HER data that included clinical, ECG, and TTE data, and achieved an excellent specificity of 0.99 and sensitivity of 0.39, with positive and negative likelihood ratios of 32 and 0.68 [78]. This text-mining algorithm, if worked in conjunction with AI-ECG models, could provide an avenue for more accurate screening for HCM.

## 4. Future Directions

Further investigation into AI’s efficacy for screening of structural heart disease is required, with more prospective investigations into its efficacy and utility still needed. Ischemic heart disease has had a significant amount of work involved, with the initial data promising. However, further investigation is still needed to improve model development and to determine its efficacy. Future research should utilize data sets with a larger population diversity for training and validation, and ideally train models utilizing many ECGs with angiographically confirmed OMI for training. Utilizing angiographically confirmed ECGs rather than only guideline-dependent ECG criteria will allow downstream re-mapping to new guideline labels with recalibration and threshold updates, rather than rebuilding models from scratch. Practically, this supports “versioned” deployment (tracked model updates), periodic recalibration, and explicit reporting of performance under multiple labeling schemes (classic STEMI criteria vs. OMI definitions) with the same data set, which may improve longevity and interpretability for clinicians. This could provide not only improved detection of OMI, but also aid clinicians in incorporating new subtle patterns to their own pattern recognition.

Kalmady et al.’s retrospective investigation into AI-ECG diagnostic performance in diagnosing fifteen cardiovascular diseases suggests models may be able to be broadened beyond one cardiovascular condition as performance seems similar to other models trained for one condition [70]. The similar performance seen in Kalmady et al.’s model versus other models may indicate that AI-ECG models without other data inputs have a limit in their performance [70]. It is still unknown whether performance is hindered by training a model to diagnose multiple cardiovascular conditions versus having models for each cardiovascular condition, and this should be investigated. Regardless, future model development should focus on incorporating more clinical data into AI-ECG models as it may aid in improving diagnostics of AI models.

Given the consistent variability across regional cohorts and the risk of bias as a first-order failure mode, the field would benefit from a shared, intentionally diverse, multicenter ECG repository paired with echocardiographic and angiographic data. Key hurdles include standardizing ECG formats across vendors, standardizing label definitions and reference standards, ensuring robust de-identification and data governance across jurisdictions, and addressing consent/ownership questions (particularly when models may later be commercialized).

The future of AI in cardiology is set to advance from retrospective analysis to real-time integration and clinical application. However, a future study should be a prospective multicenter cluster-randomized trial across diverse EMS/ED systems comparing AI-ECG-assisted triage with integrated cath lab activation versus standard care. The primary endpoint should include a hard clinical outcome (e.g., 30-day mortality or a 30–90-day composite of death, cardiogenic shock, or heart failure hospitalization), with prespecified safety endpoints such as false activations, unnecessary transfers/procedures, bleeding and contrast-associated kidney injury, and alert burden. Subgroup analyses would be essential to evaluate equity, performance across demographic strata, and generalizability across care environments.

Importantly, given the current evidence base, such a trial may be most appropriately powered for non-inferiority to standard of care, rather than expecting superiority in clinical endpoints. Instead, the primary advantages of implementation may manifest in system-level process outcomes, including faster time-to-cath-lab activation, reduced reliance on cardiology over-reads, decreased diagnostic uncertainty among frontline physicians, and increased confidence in early activation by residents and fellows. These workflow improvements could translate into more consistent recognition of OMI, fewer missed cases, and improved patient stratification; benefits that may not immediately appear as mortality reduction but could meaningfully improve real-world performance and throughput. Adoption at scale could also reduce cognitive load in busy ED settings and standardize activation thresholds, addressing the variability that currently exists across institutions and between clinicians.

AI systems may open the possibility for continuous, patient-specific monitoring and early illness identification before symptoms appear, as models develop the ability to handle data from ECGs, ECHO, cardiac CT/MRI, and EHRs all at once. It is predicted that this convergence of multimodal data will produce precision models that can predict trends like arrhythmia onset or decompensation in heart failure [21].

## 5. Limitations

This review and the underlying evidence base have several important limitations that clinicians should recognize. Most AI-ECG models were developed using retrospective data sets, single-center cohorts, or registries with enriched disease prevalence and tightly controlled ECG-reference-test intervals [34,37,38,39]. Many studies emphasize process metrics—door-to-balloon time, diagnostic accuracy, or alert latency—rather than hard outcomes such as mortality, readmission, or progression of heart failure [37,40]. Reference standards vary substantially across cohorts (expert ECG adjudication vs. coronary angiography vs. TTE with different EF thresholds), limiting comparability among models and preventing quantitative synthesis. Publication bias likely favors technically successful algorithms, so this review should be interpreted as a technical and conceptual map rather than a definitive ranking of tools.

Generalizability and spectrum bias also restrict clinical adoption. Model performance frequently declines when transitioning from tertiary centers to community or screening populations with lower disease prevalence, causing sharp reductions in positive predictive value and requiring recalibration [50,51,52]. External validations in Russia, Germany, and multinational HCM cohorts highlight how regional population characteristics, ECG acquisition practices, and disease spectrum influence performance [52,58,77]. In addition, several models show blind spots in atrial fibrillation, left-bundle branch block, paced rhythms, post-cardiac arrest ECGs, subendocardial ischemia, Takotsubo cardiomyopathy, and broad QRS complexes; contexts already challenging for clinicians [31,39,57]. Important demographic groups remain underrepresented in many training data sets, raising concerns about equity and subgroup miscalibration [53,54,55,56]. A further barrier is the paradigm shift from STEMI/NSTEMI to the occlusive MI (OMI) framework. If clinical practice continues to adopt OMI-based diagnostics, existing STEMI-focused AI models will require retraining and threshold recalibration, since algorithms built on legacy STEMI criteria may misclassify occlusions or underperform under updated diagnostic standards [42].

Finally, real-world implementation and interpretability remain early. Most deployment studies come from single systems with robust informatics support, custom alert pathways, and controlled activation protocols, limiting generalizability to diverse hospital environments [37,38]. Evidence remains limited on how AI-ECG integrates with different ECG vendors, heterogeneous EHR infrastructures, or variable catheterization activation workflows. Operational challenges including alarm fatigue, discordance between AI output and clinician judgment, responsibility for AI-triggered decisions, and medico-legal accountability are largely unaddressed. Although visualization methods such as Grad-CAM illustrate that AI models rely on nuanced waveform segments, these insights rarely translate into human-interpretable diagnostic criteria or clinician education [41]. Flagging specific waveforms that triggered an alert and reporting both a confidence level percentage and an uncertainty level could enhance usability and clinician trust. Minimum criteria for acting on AI-ECG alerts will vary per institution but should include external validation in cohorts resembling the intended deployment population, subgroup performance reporting in known “blind spot” rhythms, and calibration and threshold tuning to the local prevalence to avoid dramatic PPV collapse in screening settings. In addition, prior to full deployment, institutions should run at least a retrospective validation to estimate false alert burden and missed cases, and then implement a governance plan with continuous performance monitoring and periodic recalibration. Taken together, these limitations indicate that AI-ECG should function as an adjunct to, not a replacement for, expert clinical assessment, and that local validation, transparent subgroup reporting, and thoughtful implementation strategies must precede broad deployment.

## Figures and Tables

**Table 1:** Summary of performances of multiple AI-ECG models for detecting ischemic heart disease.

Study	Data/Setting	Model	Reference Standard	Performance	Additional Observations	Notes
Baseline computerized algorithms	Mixed literature	Rule-based commercial ECG	Varies	Sensitivity 0.62–0.93; Specificity 0.89–0.99	—	Wide performance range across products/thresholds
AI-ECG STEMI detection (Chang)	Internal and external test set	LSTM 12-lead ECG	No cath	AUROC 0.98; Cardiologist 0.898; EM 0.820; IM 0.765; Commercial 0.845	—	Limited STEMI diversity; no angiography
AI-ECG STEMI ED Triage	Single center (China Medical University Hospital)	LSTM 12-lead ECG	Three board-certified cardiologists	AUROC 0.999; Sensitivity 0.977; Specificity 0.998	Door-to-balloon time ↓ 17% (64.5 ± 35.3 min to 53.2 ± 12.7 min)	Single center Limited STEMI diversity
Prehospital STEMI triage (Chen)	Central Taiwan ambulance service	CNN + LSTM single-piece 12-lead mini-ECG	Cath (10/362)	AUROC 0.914; Accuracy 0.992; Precision 0.889; Specificity 0.994	AI 37 ± 11 s vs. MD 113 ± 369 s; some >10 min	Small STEMI number; regional; needs larger studies
ARISE RCT (Lin et al.)	Tri-Service General Hospital (Taipei, Taiwan)	AI-ECG triage	Standard of care— clinical outcomes	NPV 99.9%; PPV 89.5%	Door-to-balloon time ↓ 17% (14 min) (96 to 82 min; *p* = 0.001) ECG-to-balloon time ↓ 5.6 min (83.6 to 78 min; *p* < 0.001) cardiac death OR 0.73 (*p* = 0.029)	Single-center design, needs larger multicenter RCTs, short follow-up period Lack of evaluation of care appropriateness and clinical safety endpoints
STEMI/NSTEMI discrimination (Gustafsson)	Stockholm ~492,000 ECGs ~214,000 patients	Deep CNN/DNN 12-lead	Registry	STEMI/NSTEMI C-stat 0.991/0.832; Brier 0.001/0.008	—	STEMI characteristics: down-sloping late T wave NSTEMI characteristics: late PQ and late T wave
Queen of Hearts AI Model OMI vs. STEMI criteria (Herman)	International 2222 patients	2.5 s 12-lead AI-ECG	Coronary angiography	Sensitivity 80.6% vs. 32.5%; Specificity 93.7% vs. 97.7%; Accuracy 90.9%; AUC 0.938 vs. 0.651	Diagnosis time: AI 2.3 h; STEMI rules 5.3 h; ECG experts 2.9 h Acc 90.9%	Retrospective; waveforms only; strong external performance Lower sensitivity in LBBB and broad QRS

Abbreviations: AI-ECG: artificial intelligence-enhanced electrocardiography; AUROC: area under receiver operator curve; cath: cardiac catheterization; CNN: convolutional neural network; ECG: electrocardiography; EM: emergency medicine; IM: internal medicine; NSTEMI: non-ST-elevation myocardial infarction; NPV: negative predictive value; OR: odds ratio; PPV: positive predictive value; RCT: randomized clinical trial; STEMI: ST-elevation myocardial infarction; NSTEMI: non-ST-elevation myocardial infarction; OMI: occlusive myocardial infarction; ↓: decreased.

**Table 2:** Performance of AI-ECG and EHR-based models for detecting left ventricular dysfunction.

Study	Data/Setting	Model	Reference Standard	Performance	Additional Observations	Notes
Mayo Clinic External validation (Attia)	16,056 adults 3874 with TTE and ECG < 1 month apart Prevalence: 7.8%	CNN AI model	TTE EF	AUROC 0.918; Accuracy 86.5%; Sensitivity 82.5%; Specificity 86.8%	pro-BNP ≥125 → <5 FPs; no loss of TPs	Biomarker + AI improves precision
Know Your Heart Study External Validation (Attia)	4277 adults General population (Arkhangelsk/Novosibirsk) Prevalence: 0.6%	Attia CNN AI model	TTE EF	**AUROC 0.82** *Original Cut-off:* Sens 26.9%; Spec 97.4% *Optimized Cut-off:* Sens 84.6%; Spec 64.2% PPV 1.4–5.9%	PPV significantly decreased due to low prevalence of LVD Original cut-off failed sensitivity in screening population	
Generalizability and bias (Yagi)	Brigham and Women’s Hospital; Massachusetts General Hospital UCSF; Keio	4 de novo AI-ECGs	TTE EF	MGH AUROC 0.914/0.905; Keio 0.914/0.856	—	Performance varies by cohort ↓ performance in AF/LBBB/paced
European external validation (König)	Germany	Yagi model	TTE EF	AUROC 0.88; Sensitivity 82%; Specificity 77%; NPV 96%	False-positive HR ~ 4 for future LVSD	Population-specific training needed

Abbreviations: AF: atrial fibrillation; AI: artificial intelligence; AI-ECG: artificial intelligence-enhanced electrocardiography; AUROC: area under receiver operator curve; CNN: convolutional neural network; ECG: electrocardiography; EF: ejection fraction; FP: false positive; HR: hazard ratio; LBBB: left-bundle branch block; LVSD: left ventricular systolic dysfunction; MGH: Massachusetts General Hospital; NPV: negative predictive value; TP: true positive; TTE: transthoracic echocardiogram; UCSF: University of California, San Francisco; →: lead to; ↓: decreased.

**Table 3:** Summary of performances of multiple AI-ECG models and AI-ECG with EHR-based models for detecting structural heart disease.

Study	Data/Setting	Model	Reference Standard	Performance	Additional Observations	Notes
Moderate–severe aortic stenosis (Cohen-Shelly)	129,788 training; 25,893 validation; 102,926 test	AI-ECG ± age/sex	TTE	AUC 0.85; Sensitivity 78%; Specificity 74%; Accuracy 74%; +age/sex AUC 0.87	FP HR 2.18 @15y	Similar risk signal across MR/AR/AS/TR/PR
ECG-based HCM detection (Ko/Attia)	Mayo Clinic: 46,901 training; 6700 validation; 13,400 testing	CNN AI-ECG	TTE/cMRI ± clinical	Internal AUC 0.96; Sensitivity 87% (95% in young patients); Specificity 90% (92% in young patients)	—	Improved performance in young patient population
Ko/Attia external validation (Siontis)	Switzerland, UK, South Korea (external validation) 773 HCM patients; 3867 controls	—	ESC/ACC/AHA imaging criteria and cardiologist adjudication	External AUC 0.92; Accuracy 86.9%; Sensitivity 82.8%; Specificity 87.7%	—	Only 2.2% of patients were Black Requires prospective screening trials
EHR text-mining triage (Sammani)	—	ML text-mining (EHR + ECG + TTE)	Clinical adjudication	Sensitivity 0.39; Specificity 0.99; LR+ 32; LR− 0.68	Efficient rule-in prefilter	Best as complement; ↑ PPV/workflow

Abbreviations: ACC: American College of Cardiology; AHA: American Heart Association; AI-ECG: artificial intelligence-enabled electrocardiography; AR: aortic regurgitation; AS: aortic stenosis; AUC: area under the curve (receiver operating characteristic); cMRI: cardiac magnetic resonance imaging; CNN: convolutional neural network; ECG: electrocardiography; EHR: electronic health record; ESC: European Society of Cardiology; FP: false positive; HCM: hypertrophic cardiomyopathy; HR: hazard ratio; LR+: positive likelihood ratio; LR−: negative likelihood ratio; ML: machine learning; MR: mitral regurgitation; PPV: positive predictive value; PR: pulmonic regurgitation; TR: tricuspid regurgitation; TTE: transthoracic echocardiogram; UK: United Kingdom; ↑: increased.

## Data Availability

No new data were created or analyzed in this study. Data sharing is not applicable to this article.

## Abbreviations

The following abbreviations are used in this manuscript:

ACC	American College of Cardiology
ACS	Acute Coronary Syndrome
AF	Atrial fibrillation
AHA	American Heart Association
AI	Artificial intelligence
AI-ECG	Artificial intelligence-enabled Electrocardiography
AI-STEMI	Artificial intelligence–based STEMI detection model
AR	Aortic regurgitation
ARISE	Artificial Intelligence-Powered Rapid Identification of ST-Elevation Myocardial Infarction via Electrocardiogram
AS	Aortic stenosis
AUC	Area under the curve
AUROC	Area under the receiver operating characteristic curve
BNP	B-type natriuretic peptide
BWH	Brigham and Women’s Hospital
C-stat	Concordance statistic
CCL	Cardiac catheterization laboratory
CI	Confidence interval
CMR	Cardiac magnetic resonance
CNN	Convolutional neural network
CNN-LSTM	Convolutional neural network–long short-term memory hybrid model
CT	Computed tomography
CVD	Cardiovascular disease
cMRI	Cardiac magnetic resonance imaging
DL	Deep learning
DNN	Deep neural network
Dx	Diagnosis
ECG	Electrocardiogram
ED	Emergency Department
EF	Ejection fraction
ECHO	Echocardiography
EHR	Electronic health record
ESC	European Society of Cardiology
EVOLVED	Early intervention versus conservative management in asymptomatic severe aortic stenosis with myocardial fibrosis
FP	False positive
Grad-CAM	Gradient-weighted class activation mapping
HCM	Hypertrophic cardiomyopathy
HR	Hazard ratio
IHD	Ischemic heart disease
Keio	Keio University Hospital
LBBB	Left-bundle branch block
LR+	Positive likelihood ratio
LR−	Negative likelihood ratio
LSTM	Long short-term memory
LVD	Left ventricular dysfunction
LVSD	Left ventricular systolic dysfunction
LVEF	Left ventricular ejection fraction
MGH	Massachusetts General Hospital
MI	Myocardial infarction
ML	Machine learning
MRI	Magnetic resonance imaging
MR	Mitral regurgitation
NPV	Negative predictive value
NSTEMI	Non-ST-elevation myocardial infarction
NYHA	New York Heart Association
OMI	Occlusive myocardial infarction
Ops	Operational characteristics/notes
OR	Odds ratio
PPV	Positive predictive value
PR	Pulmonic regurgitation
pro-BNP	N-terminal pro-B-type natriuretic peptide
pts	Patients
QRS	QRS complex
QT	QT interval
RCT	Randomized clinical trial
Ref Std	Reference standard
RNN	Recurrent neural network
Sens	Sensitivity
SOC	Standard of care
Spec	Specificity
STEMI	ST-elevation myocardial infarction
TP	True positives
TR	Tricuspid regurgitation
TTE	Transthoracic echocardiography
UCSF	University of California, San Francisco
US	United States
VHD	Valvular heart disease
